# Supplementary material for: HIVprotI: an integrated web based platform for prediction and design of HIV proteins inhibitors
Source: J Cheminform. 2018 Mar 9;10:12. doi: 10.1186/s13321-018-0266-y (PMC5845081; doi:10.1186/s13321-018-0266-y)
Supplement: Supplementary file 2 — Additional file 2. Source code of HIVProtI web server. [file 13321_2018_266_MOESM2_ESM.zip › HIVprotI_Source-code/hivprotI/weka-3-6-6/doc/weka/classifiers/trees/lmt/LMTNode.html]

LMTNode


---


|  |  |  |
| --- | --- | --- |
| **Overview** \| **Package** \| **Class** \| **Tree** \| **Deprecated** \| **Index** \| **Help** \| **Weka's home** | |  |
| PREV CLASS   **NEXT CLASS** | **FRAMES**    **NO FRAMES**     **All Classes** |  |
| SUMMARY: NESTED \| FIELD \| CONSTR \| METHOD | DETAIL: FIELD \| CONSTR \| METHOD |  |


---


## weka.classifiers.trees.lmt Class LMTNode

```
java.lang.Object
  weka.classifiers.Classifier
      weka.classifiers.trees.lmt.LogisticBase
          weka.classifiers.trees.lmt.LMTNode
```

**All Implemented Interfaces:**: java.io.Serializable, java.lang.Cloneable, CapabilitiesHandler, OptionHandler, RevisionHandler, WeightedInstancesHandler

---

``` public class LMTNode extends LogisticBase ```

Class for logistic model tree structure.

**Version:**
:   $Revision: 1.8 $

**Author:**
:   Niels Landwehr, Marc Sumner

**See Also:**: Serialized Form

---

| **Field Summary** | |
| --- | --- |
| `double` | `m_alpha`             Alpha-value (for pruning) at the node |
| `double` | `m_numIncorrectModel`             Weighted number of training examples currently misclassified by the logistic model at the node |
| `double` | `m_numIncorrectTree`             Weighted number of training examples currently misclassified by the subtree rooted at the node |


**Constructor Summary**

`LMTNode(ModelSelection modelSelection,
int numBoostingIterations,
boolean fastRegression,
boolean errorOnProbabilities,
int minNumInstances,
double weightTrimBeta,
boolean useAIC)`
  
          Constructor for logistic model tree node.


| **Method Summary** | |
| --- | --- |
| `int` | `assignIDs(int lastID)`             Assigns unique IDs to all nodes in the tree |
| `int` | `assignLeafModelNumbers(int leafCounter)`             Assigns numbers to the logistic regression models at the leaves of the tree |
| `void` | `buildClassifier(Instances data)`             Method for building a logistic model tree (only called for the root node). |
| `void` | `buildTree(Instances data, SimpleLinearRegression[][] higherRegressions, double totalInstanceWeight, double higherNumParameters)`             Method for building the tree structure. |
| `void` | `calculateAlphas()`             Updates the alpha field for all nodes. |
| `void` | `cleanup()`             Cleanup in order to save memory. |
| `double[]` | `distributionForInstance(Instance instance)`             Returns the class probabilities for an instance given by the logistic model tree. |
| `java.lang.String` | `getModelParameters()`             Returns a string describing the number of LogitBoost iterations performed at this node, the total number of LogitBoost iterations performed (including iterations at higher levels in the tree), and the number of training examples at this node. |
| `java.util.Vector` | `getNodes()`             Return a list of all inner nodes in the tree |
| `void` | `getNodes(java.util.Vector nodeList)`             Fills a list with all inner nodes in the tree |
| `int` | `getNumInnerNodes()`             Method to count the number of inner nodes in the tree |
| `int` | `getNumLeaves()`             Returns the number of leaves in the tree. |
| `java.lang.String` | `getRevision()`             Returns the revision string. |
| `java.lang.String` | `graph()`             Returns graph describing the tree. |
| `boolean` | `hasModels()`             Returns true if the logistic regression model at this node has changed compared to the one at the parent node. |
| `double[]` | `modelDistributionForInstance(Instance instance)`             Returns the class probabilities for an instance according to the logistic model at the node. |
| `void` | `modelErrors()`             Updates the numIncorrectModel field for all nodes. |
| `java.lang.String` | `modelsToString()`             Returns a string describing the logistic regression function at the node. |
| `int` | `numLeaves()`             Returns the number of leaves (normal count). |
| `int` | `numNodes()`             Returns the number of nodes. |
| `void` | `prune(double alpha)`             Prunes a logistic model tree using the CART pruning scheme, given a cost-complexity parameter alpha. |
| `int` | `prune(double[] alphas, double[] errors, Instances test)`             Method for performing one fold in the cross-validation of the cost-complexity parameter. |
| `java.lang.String` | `toString()`             Returns a description of the logistic model tree (tree structure and logistic models) |
| `void` | `treeErrors()`             Updates the numIncorrectTree field for all nodes. |

**Methods inherited from class weka.classifiers.trees.lmt.LogisticBase**

`getMaxIterations, getNumRegressions, getUseAIC, getUsedAttributes, getWeightTrimBeta, percentAttributesUsed, setHeuristicStop, setMaxIterations, setUseAIC, setWeightTrimBeta`

**Methods inherited from class weka.classifiers.Classifier**

`classifyInstance, debugTipText, forName, getCapabilities, getDebug, getOptions, listOptions, makeCopies, makeCopy, setDebug, setOptions`

**Methods inherited from class java.lang.Object**

`equals, getClass, hashCode, notify, notifyAll, wait, wait, wait`

**Field Detail**

### m\_alpha

```
public double m_alpha
```

:   Alpha-value (for pruning) at the node

---


### m\_numIncorrectModel

```
public double m_numIncorrectModel
```

:   Weighted number of training examples currently misclassified by the logistic model at the node

---


### m\_numIncorrectTree

```
public double m_numIncorrectTree
```

:   Weighted number of training examples currently misclassified by the subtree rooted at the node


**Constructor Detail**

### LMTNode

```
public LMTNode(ModelSelection modelSelection,
               int numBoostingIterations,
               boolean fastRegression,
               boolean errorOnProbabilities,
               int minNumInstances,
               double weightTrimBeta,
               boolean useAIC)
```

:   Constructor for logistic model tree node.

    **Parameters:**: `modelSelection` - selection method for local splitting model: `numBoostingIterations` - sets the numBoostingIterations parameter: `fastRegression` - sets the fastRegression parameter: `errorOnProbabilities` - Use error on probabilities for stopping criterion of LogitBoost?: `minNumInstances` - minimum number of instances at which a node is considered for splitting


**Method Detail**

### buildClassifier

```
public void buildClassifier(Instances data)
                     throws java.lang.Exception
```

:   Method for building a logistic model tree (only called for the root node).
    Grows an initial logistic model tree and prunes it back using the CART pruning scheme.

    :   **Overrides:**: `buildClassifier` in class `LogisticBase`
    :   **Parameters:**: `data` - the data to train with **Throws:**: `java.lang.Exception` - if something goes wrong

---


### buildTree

```
public void buildTree(Instances data,
                      SimpleLinearRegression[][] higherRegressions,
                      double totalInstanceWeight,
                      double higherNumParameters)
               throws java.lang.Exception
```

:   Method for building the tree structure.
    Builds a logistic model, splits the node and recursively builds tree for child nodes.

    :   **Parameters:**: `data` - the training data passed on to this node: `higherRegressions` - An array of regression functions produced by LogitBoost at higher levels in the tree. They represent a logistic regression model that is refined locally at this node.: `totalInstanceWeight` - the total number of training examples: `higherNumParameters` - effective number of parameters in the logistic regression model built in parent nodes **Throws:**: `java.lang.Exception` - if something goes wrong

---


### prune

```
public void prune(double alpha)
           throws java.lang.Exception
```

:   Prunes a logistic model tree using the CART pruning scheme, given a
    cost-complexity parameter alpha.

    :   **Parameters:**: `alpha` - the cost-complexity measure **Throws:**: `java.lang.Exception` - if something goes wrong

---


### prune

```
public int prune(double[] alphas,
                 double[] errors,
                 Instances test)
          throws java.lang.Exception
```

:   Method for performing one fold in the cross-validation of the cost-complexity parameter.
    Generates a sequence of alpha-values with error estimates for the corresponding (partially pruned)
    trees, given the test set of that fold.

    :   **Parameters:**: `alphas` - array to hold the generated alpha-values: `errors` - array to hold the corresponding error estimates: `test` - test set of that fold (to obtain error estimates) **Throws:**: `java.lang.Exception` - if something goes wrong

---


### getNumInnerNodes

```
public int getNumInnerNodes()
```

:   Method to count the number of inner nodes in the tree

    :   **Returns:**: the number of inner nodes

---


### getNumLeaves

```
public int getNumLeaves()
```

:   Returns the number of leaves in the tree.
    Leaves are only counted if their logistic model has changed compared to the one of the parent node.

    :   **Returns:**: the number of leaves

---


### modelErrors

```
public void modelErrors()
                 throws java.lang.Exception
```

:   Updates the numIncorrectModel field for all nodes. This is needed for calculating the alpha-values.

    :   **Throws:**: `java.lang.Exception`

---


### treeErrors

```
public void treeErrors()
```

:   Updates the numIncorrectTree field for all nodes. This is needed for calculating the alpha-values.

---


### calculateAlphas

```
public void calculateAlphas()
                     throws java.lang.Exception
```

:   Updates the alpha field for all nodes.

    :   **Throws:**: `java.lang.Exception`

---


### getNodes

```
public java.util.Vector getNodes()
```

:   Return a list of all inner nodes in the tree

    :   **Returns:**: the list of nodes

---


### getNodes

```
public void getNodes(java.util.Vector nodeList)
```

:   Fills a list with all inner nodes in the tree

    :   **Parameters:**: `nodeList` - the list to be filled

---


### hasModels

```
public boolean hasModels()
```

:   Returns true if the logistic regression model at this node has changed compared to the
    one at the parent node.

    :   **Returns:**: whether it has changed

---


### modelDistributionForInstance

```
public double[] modelDistributionForInstance(Instance instance)
                                      throws java.lang.Exception
```

:   Returns the class probabilities for an instance according to the logistic model at the node.

    :   **Parameters:**: `instance` - the instance **Returns:**: the array of probabilities **Throws:**: `java.lang.Exception`

---


### distributionForInstance

```
public double[] distributionForInstance(Instance instance)
                                 throws java.lang.Exception
```

:   Returns the class probabilities for an instance given by the logistic model tree.

    :   **Overrides:**: `distributionForInstance` in class `LogisticBase`
    :   **Parameters:**: `instance` - the instance **Returns:**: the array of probabilities **Throws:**: `java.lang.Exception` - if distribution can't be computed successfully

---


### numLeaves

```
public int numLeaves()
```

:   Returns the number of leaves (normal count).

    :   **Returns:**: the number of leaves

---


### numNodes

```
public int numNodes()
```

:   Returns the number of nodes.

    :   **Returns:**: the number of nodes

---


### toString

```
public java.lang.String toString()
```

:   Returns a description of the logistic model tree (tree structure and logistic models)

    :   **Overrides:**: `toString` in class `LogisticBase`
    :   **Returns:**: describing string

---


### getModelParameters

```
public java.lang.String getModelParameters()
```

:   Returns a string describing the number of LogitBoost iterations performed at this node, the total number
    of LogitBoost iterations performed (including iterations at higher levels in the tree), and the number
    of training examples at this node.

    :   **Returns:**: the describing string

---


### assignIDs

```
public int assignIDs(int lastID)
```

:   Assigns unique IDs to all nodes in the tree

---


### assignLeafModelNumbers

```
public int assignLeafModelNumbers(int leafCounter)
```

:   Assigns numbers to the logistic regression models at the leaves of the tree

---


### modelsToString

```
public java.lang.String modelsToString()
```

:   Returns a string describing the logistic regression function at the node.

---


### graph

```
public java.lang.String graph()
                       throws java.lang.Exception
```

:   Returns graph describing the tree.

    :   **Throws:**: `java.lang.Exception` - if something goes wrong

---


### cleanup

```
public void cleanup()
```

:   Cleanup in order to save memory.

    :   **Overrides:**: `cleanup` in class `LogisticBase`

---


### getRevision

```
public java.lang.String getRevision()
```

:   Returns the revision string.

    :   **Specified by:**: `getRevision` in interface `RevisionHandler` **Overrides:**: `getRevision` in class `LogisticBase`
    :   **Returns:**: the revision


---


|  |  |  |
| --- | --- | --- |
| **Overview** \| **Package** \| **Class** \| **Tree** \| **Deprecated** \| **Index** \| **Help** \| **Weka's home** | |  |
| PREV CLASS   **NEXT CLASS** | **FRAMES**    **NO FRAMES**     **All Classes** |  |
| SUMMARY: NESTED \| FIELD \| CONSTR \| METHOD | DETAIL: FIELD \| CONSTR \| METHOD |  |


---
